# Production and glyco-engineering of immunomodulatory helminth glycoproteins in plants

**DOI:** 10.1038/srep45910

**Published:** 2017-04-10

**Authors:** Ruud H. P. Wilbers, Lotte B. Westerhof, Kim van Noort, Katja Obieglo, Nicole N. Driessen, Bart Everts, Sonja I. Gringhuis, Gabriele Schramm, Aska Goverse, Geert Smant, Jaap Bakker, Hermelijn H. Smits, Maria Yazdanbakhsh, Arjen Schots, Cornelis H. Hokke

**Affiliations:** 1 Laboratory of Nematology, Plant Sciences Group, Wageningen University and Research, Droevendaalsesteeg 1, 6708 PB Wageningen, The Netherlands; 2 Department of Parasitology, Leiden University Medical Center, Albinusdreef 2, 2333 ZA Leiden, The Netherlands; 3 Department of Experimental Immunology, Academic Medical Center, University of Amsterdam, Meibergdreef 9, 1105 AZ Amsterdam, The Netherlands; 4 Research Center Borstel, Priority Area Asthma and Allergy, Experimental Pneumology, Parkallee 22, D-23845, Borstel, Germany

## Abstract

Helminth parasites control host-immune responses by secreting immunomodulatory glycoproteins. Clinical trials and mouse model studies have demonstrated the potential of helminth-derived glycoproteins for the treatment of immune-related diseases, like allergies and autoimmune diseases. Studies are however hampered by the limited availability of native parasite-derived proteins. Moreover, recombinant protein production systems have thus far been unable to reconstitute helminth-like glycosylation essential for the functionality of some helminth glycoproteins. Here we exploited the flexibility of the N-glycosylation machinery of plants to reconstruct the helminth glycoproteins omega-1 and kappa-5, two major constituents of immunomodulatory *Schistosoma mansoni* soluble egg antigens. Fine-tuning transient co-expression of specific glycosyltransferases in *Nicotiana benthamiana* enabled the synthesis of Lewis X (LeX) and LDN/LDN-F glycan motifs as found on natural omega-1 and kappa-5, respectively. *In vitro* and *in vivo* evaluation of the introduction of native LeX motifs on plant-produced omega-1 confirmed that LeX on omega-1 contributes to the glycoprotein’s Th2-inducing properties. These data indicate that mimicking the complex carbohydrate structures of helminths in plants is a promising strategy to allow targeted evaluation of therapeutic glycoproteins for the treatment of inflammatory disorders. In addition, our results offer perspectives for the development of effective anti-helminthic vaccines by reconstructing native parasite glycoprotein antigens.

Parasitic helminths such as schistosomes or hookworms achieve long-term infection and survival by actively modulating the immune system of their host^1^,^2^. The immunomodulatory properties of helminths are mediated for a large part by their secretory glycoproteins, which induce T helper cell type-2 (Th2) immunity and regulatory networks via glycan-dependent mechanisms^3^,^4^,^5^,^6^. Defined secretory glycoproteins of helminths therefore could form a novel class of biopharmaceuticals with great potential for the treatment of allergic conditions, autoimmune diseases, metabolic syndrome and other inflammatory disorders^7^,^8^,^9^,^10^. Although helminth glycans are highly diverse, they do display typical characteristics such as the lack of sialylation, a high proportion of fucosylation, the possible occurrence of the plant-like N-glycan core modifications β1,2-xylose and α1,3-fucose, and a high abundance of antennary GalNAcβ1-4GlcNAc (LDN). The core-modified N-glycans, as well as N-glycans that carry Galβ1-4(Fucα1-3)GlcNAc (LeX), GalNAcβ1-4(Fucα1-3)GlcNAc (LDN-F) and LDN have all been identified as functional elements of immunomodulatory helminth glycoproteins^11^. To support fundamental studies of the mechanisms of helminth glycoprotein-induced immune modulation and the exploration of helminth glycoproteins in clinical models, a recombinant production platform is required. For such a platform the controlled production of pure and defined recombinant helminth glycoproteins with functional glycosylation is essential.

In the last two decades, plants have emerged as a versatile expression platform for the production of recombinant proteins^12^. This particularly holds for the expression of glycoproteins, as plants are highly compliant to engineered adaptations of their endogenous N-glycosylation machinery^13^. Also, in contrast to other production platforms such as yeasts or CHO and HEK cells, plant-produced glycoproteins are remarkably homogeneous in their N-glycan composition^13^,^14^. Interestingly, some of the typical characteristics of the limited plant glycome match those of helminths, including the non-mammalian N-glycan core modifications and the lack of sialylation. All these characteristics together inspired us to investigate if plants can be used to produce biologically active helminth glycoproteins with a defined and tailored N-glycan composition.

Here, we present a platform for the fast, transient expression of large amounts of helminthized recombinant glycoproteins in leaves of *Nicotiana benthamiana* plants. We demonstrated the versatility of our method by producing two functionally and structurally distinct *Schistosoma mansoni* egg glycoproteins, kappa-5 and omega-1. Kappa-5 is an egg component that carries the LDN and LDN-F motifs, which are implicated in granuloma formation and immunomodulation, respectively^15^. Omega-1 is the major, glycan-dependent Th2-polarizing compound of *S. mansoni* eggs^4^,^16^ and it carries LeX motifs on its N-glycans. Furthermore, the immunomodulatory functionalities of plant-produced omega-1 with and without the LeX motif were assessed *in vitro* and *in vivo* to confirm the bioactivity of the glycoprotein as well as the critical contribution of properly engineered glycosylation.

## Results and Discussion

### Helminth glycoproteins are efficiently produced in *N. benthamiana*

**Figure 1:**
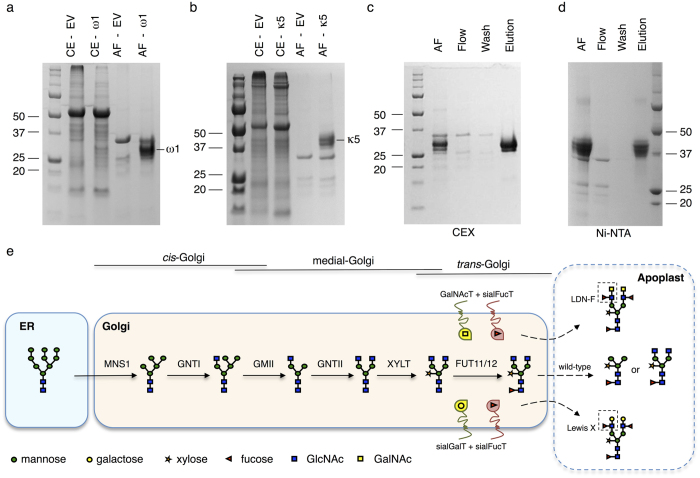
Plant-based production of helminth glycoproteins with tailored N-glycans. (**a-b**) SDS-PAGE and Coomassie blue staining of crude extracts (CE) and apoplast fluids (AF) from omega-1 (ω1), kappa-5 (κ5) or empty vector (EV) infiltrated plants reveals efficient secretion of both omega-1 (**a**) and kappa-5 (**b**) into the leaf extracellular space (apoplast). (**c-d**) Efficient secretion enables single-step purification from the leaf apoplast fluid by cation exchange chromatography (CEX) for omega-1 (**c**) or Ni-NTA chromatography for kappa-5 (**d**). (**e**) A schematic overview of the successive N-glycan modifying steps in the plant Golgi-system. MNSI: Golgi-α-mannosidase I; GnTI: *N-*acetyl-glucosaminyltransferase I; GMII: Golgi- α-mannosidase II; GnTII: *N-*acetyl-glucosaminyltransferase II; XYLT: β1,2-xylosyltransferase; FUT11/12: core α1,3-fucosyltransferase. The plant N-glycosylation machinery was engineered by introducing (hybrid) glycosyltransferases that allow the synthesis of LeX or LDN-F motifs.

To achieve high expression levels of recombinant omega-1 and kappa-5, we applied an in-house codon optimization strategy to match the codon use of these helminth genes to highly expressed genes in plants. Expression was achieved by agroinfiltration of *Nicotiana benthamiana* plants. Accumulation of omega-1 (~30 kDa) and kappa-5 (~41 kDa) in crude extracts and apoplast fluids (leaf extracellular space) was analyzed by SDS-PAGE (Fig. 1a,b). The majority of both glycoproteins (~90%) was recovered from the apoplast, which indicates that both omega-1 and kappa-5 were secreted with remarkable efficiency. Only few endogenous plant proteins were present in these apoplast fluids, which facilitated single-step purification of >0.5 mg of omega-1 or kappa-5 per plant (3–4 gram fresh leaf material) by cation-exchange and Ni-NTA chromatography, respectively (Fig. 1c,d). The N-glycan composition of purified omega-1 and kappa-5 was assessed by matrix-assisted laser desorption/ionisation time-of-flight mass spectrometry (MALDI-TOF MS) analysis of released N-glycans. The predominant N-glycans on both proteins carry typical plant β1,2-xylose and core α1,3-fucose residues (Supplemental Fig. 1). However, omega-1 mainly displays terminal mannose residues on paucimannosidic glycans (Supplemental Fig. 1a), whereas the kappa-5 N-glycan trimannosyl core is substituted with GlcNAc residues (Supplemental Fig. 1b). So, even though both omega-1 and kappa-5 are isolated and purified from the apoplast, their N-glycan composition is strikingly different. Previously, we also observed the presence of high proportions (~50%) of Lewis A on the N-glycans of recombinant human IL-22 when isolated and purified from the apoplast of *N. benthamiana* plants^14^. These differences in N-glycan composition most likely arise from different intrinsic protein characteristics, which in the case of omega-1 and kappa-5 could be a difference in sensitivity towards endogenous β-hexosaminidase activity.

### Synthesis of LDN and LDN-F glycan motifs on kappa-5

Kappa-5 is a schistosome egg component that carries LDN and LDN-F glycan motifs^15^. Engineering of GalNAc-carrying glycans in plants has to our knowledge only been attempted for the synthesis of mammalian mucin-type O-glycans^17^,^18^,^19^. These O-glycans were previously synthesized on recombinant MUC1 in tobacco and required the co-expression of human GalNAc-T2 in combination with a UDP-GlcNAc C4-epimerase and the UDP-GalNAc transporter nstp-4^19^. Yet, more recent reports showed that it is possible to synthesize GalNAc containing O-glycans on several recombinant proteins in the absence of a transporter and/or epimerase^17^,^18^. To achieve LDN synthesis on N-glycans in plants, we tested co-expression of different combinations of a β1,4-*N-*acetylgalactosaminyltransferase (GalNAcT; Fig. 1e), UDP-GalNAc transporters (*sqv-7, nstp-4 and nstp-5*) and UDP-GlcNAc C4 epimerase along with kappa-5.

**Figure 2:**
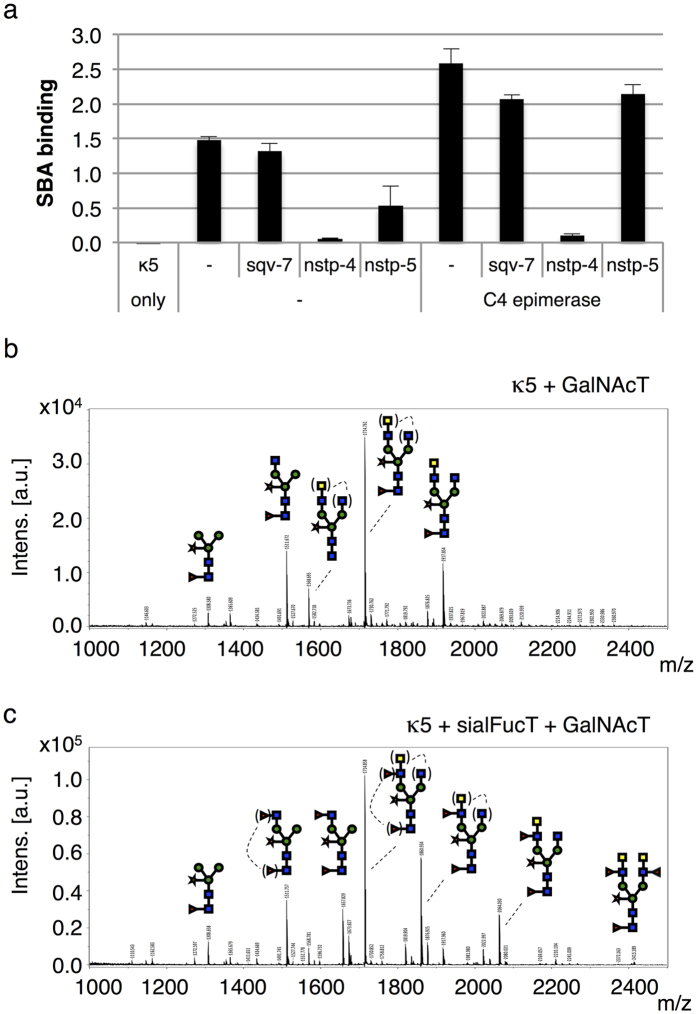
Engineering of LDN carrying N-glycans in plants. Co-expression of different combinations of native GalNAcT, UDP-GalNAc transporters (*sqv-7, nstp-4 and nstp-5*) and/or C4 epimerase was performed to determine which of these genes are required for *in planta* engineering of LDN carrying N-glycans. (**a**) Soybean agglutinin (SBA) binding assay on total soluble proteins from apoplast fluids reveals that expression of native GalNAcT from *C. elegans* is sufficient for the synthesis of LDN carrying N-glycans. (**b**) MALDI-TOF MS N*-*glycan profile for kappa-5 upon co-expression of native GalNAcT reveals the synthesis of LDN motifs. (**c**) MALDI-TOF MS N*-*glycan profile for kappa-5 upon co-expression of *trans*-Golgi-targeted sialFucT and native GalNAcT reveals the synthesis of LDN-F motifs. Sugar residues are placed between brackets when a MS peak represents multiple N-glycan structures of identical mass.

Soybean agglutinin (SBA) binding assays were used to screen for the presence of LDN carrying N-glycans on total apoplast proteins and showed that co-expression of native GalNAcT is sufficient for LDN synthesis in plants (Fig. 2a). Co-expression of the C4 epimerase increased the binding of SBA to total apoplast proteins, however increased binding to purified kappa-5 was not observed (data not shown). Strikingly, nstp4 almost completely blocks the synthesis of LDN. This could indicate that nstp-4 re-directs the UDP-GalNAc substrate to a Golgi compartment where LDN synthesis does not take place. MALDI-TOF MS analysis confirmed the presence of LDN motifs on the N-glycans released from purified kappa-5 (Fig. 2b). Approximately 30% of the isolated N-glycans from kappa-5 carried a single LDN motif as was shown by enzymatic digestion with β-*N*-acetylhexosaminidase and β-*N*-acetylglucosaminidase and subsequent MALDI-TOF MS product analysis (Supplemental Fig. 2).

Next, successful synthesis of LDN-F was achieved by co-expression of a hybrid α1,3-fucosyltransferase IXa (FucT) with kappa-5 and GalNAcT (Fig. 2c). The FucT contained the CTS domain (C, cytoplasmic tail; T, transmembrane domain; S, stem region) of rat α2,6-sialyltransferase (sial), which targets the enzyme to the *trans*-Golgi compartment^20^. Digestions with β-*N*-acetylhexosaminidase and β-*N*-acetylglucosaminidase confirmed that ~50% of the N-glycans carried a single LDN-F motif (Supplemental Fig. 3). Fucosylation of the LDN motif therefore enhances the accumulation of GalNAc containing N-glycans. It is likely that fucosylated LDN motifs are more resistant against the activity of apoplastic β-*N*-acetylhexosaminidases, which are known for their ability to remove unsubstituted GlcNAc and/or GalNAc residues^21^. A significant proportion of N-glycan structures with a fucosylated terminal GlcNAc residue was observed as well (Fig. 2c, Supplemental Fig. 3). The LeX-type FucT IXa catalyzes fucose transfer to the 3-OH group of Galβ1-4GlcNAc, but not to unsubstituted GlcNAc of N-glycans^22^. This suggests that the LeX-homologous LDN-F element must have been present on these N-glycans. GalNAc residues were most likely removed by endogenous plant β-*N*-acetylhexosaminidases upon secretion into the apoplast. This would also infer that tobacco β-*N*-acetylhexosaminidases are able to remove terminal GalNAc residues in the presence of fucose on GlcNAc, similar to Jack bean β-hexosaminidase^15^.

### Synthesis of LeX glycan motifs on omega-1

**Figure 3:**
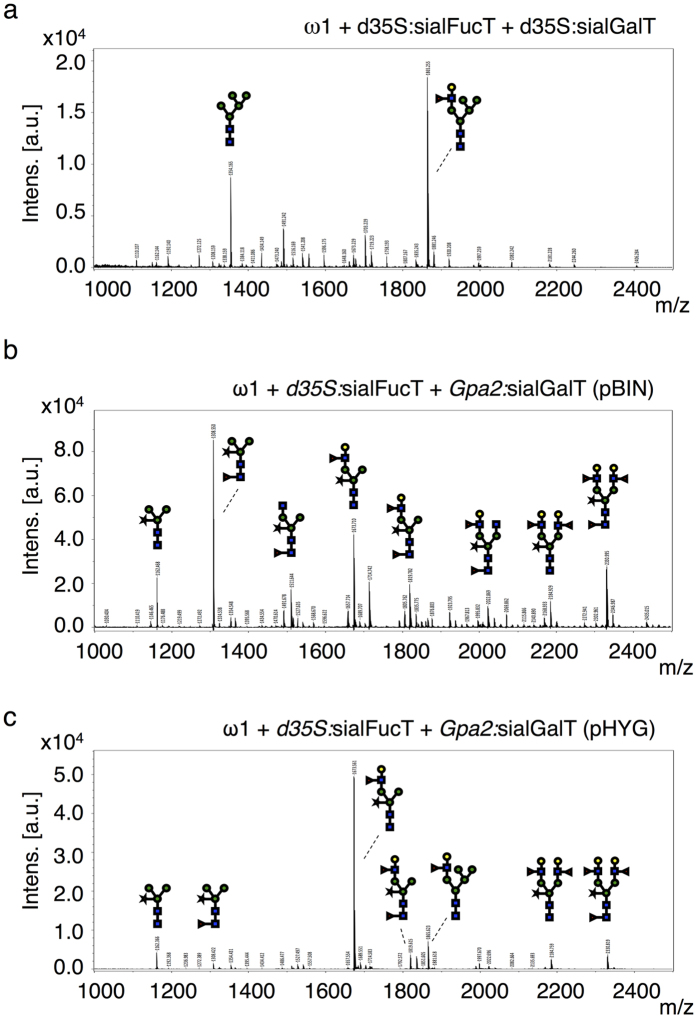
Controlled expression of sialGalT enables synthesis of non-hybrid LeX glycan motifs. Engineering of LeX on omega-1 was attempted with different expression strategies for sialGalT. N*-*glycan composition was analyzed by MALDI-TOF-MS. (**a**) N*-*glycan profile upon co-expression of *d35S:*sialFucT and *d35S:*sialGalT both in the pBIN vector. (**b**) N*-*glycan profile upon co-expression of *d35S:*sialFucT and *Gpa2:*sialGalT both in the pBIN vector. The weaker constitutive *Gpa*2 promoter was chosen to reduce sialGalT expression. (**c**) N*-*glycan profile upon co-expression of *d35S:*sialFucT and *Gpa2:*sialGalT, but the latter being expressed using the pHYG vector. The pHYG vector was chosen as it generally yields more protein compared to pBIN.

Omega-1 is a T2 ribonuclease and is one of the major Th2-polarizing compounds secreted by *S. mansoni* eggs^4^,^16^. The LeX motif, which is present on the N-glycans of native omega-1, is capable of inducing Th2 responses *in vivo*^3^ and N-glycans of omega-1 are critically involved in the functionality of the glycoprotein^4^. In order to allow synthesis of LeX glycan motifs in plants, we introduced a sialFucT and a similar hybrid for β1,4-galactosyltransferase 1 (sialGalT)^22^,^23^ into the plant N-glycosylation machinery by transient co-expression (Fig. 1e). We used the rat α2,6-sialyltransferase CTS domain instead of the medial-Golgi targeting CTS domain of xylosyltransferase previously used by Rouwendal and co-workers^22^ to avoid synthesis of hybrid LeX-type N-glycans. When a double CaMV 35 S promoter was used to drive the expression of sialGalT, we still obtained hybrid LeX-type N-glycans on omega-1 (Fig. 3a). This suggests that transient overexpression of sialGalT still interferes with the activity of endogenous glycan-modifying enzymes, like α-mannosidase II and xylosyltransferase, in the medial-Golgi (Fig. 1e). Therefore, it seems that the activity of sialGalT is not restricted to the *trans*-Golgi when the enzyme is expressed under the control of the strong dual 35 S promoter.

To reduce the expression of sialGalT we used a weaker constitutive promoter (from the potato resistance gene *Gpa2*) to drive expression of sialGalT from two different expression vectors (pBIN and pHYG). The predominant N*-*glycan type found on omega-1 upon co-expression of sialFucT and *Gpa2*:sialGalT from pBIN was the typical plant paucimannosidic N*-*glycan (Fig. 3b), but also significant proportions of monoantennary and diantennary N-glycans carrying LeX motifs were found. The pHYG expression vector, on the other hand, generally gives more mRNA transcript levels for a heterologous expressed gene most likely due to its smaller size and higher copy number in bacteria. This controlled expression of *Gpa2*:sialGalT with the pHYG vector resulted in an almost complete lack of hybrid LeX-type N*-*glycans and enabled the synthesis of relatively homogeneous N-glycans carrying a single LeX motif (Fig. 3c). The presence of LeX was confirmed by combined α(1–3,4)-fucosidase and β(1–4,6)-galactosidase treatment of the PNGase-A-released glycans and subsequent MALDI-TOF MS product analysis (Supplemental Fig. 4).

Our findings demonstrate that controlled expression of a *trans*-Golgi-targeted sialGalT is required to prevent the inhibition of endogenous mannosidase II and xylosyltransferase in the medial-Golgi. These findings also point at another layer of control of glyco-engineering, as both a proper CTS domain and quantitatively controlled expression of glycosyltransferases are required for correct localization of their biological activity in the Golgi. Similar to the synthesis of LDN/LDN-F motifs, the LeX motif is present on a single N-glycan branch. The addition of either galactose or GalNAc on the α1,3-mannosyl branch appears to still interfere with the activity of endogenous *N-*acetyl-glucosaminyltransferase II (GnTII) thereby blocking the formation of diantennary glycans to a large extent.

### Functional evaluation of plant produced omega-1

**Figure 4:**
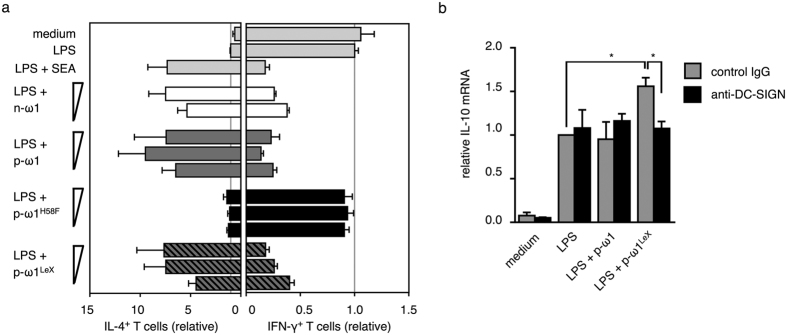
Th2 priming capacity of plant-produced omega-1. (**a**) Human monocyte-derived dendritic cells (DCs) were pulsed with indicated reagents for 48 hours, after which they were co-cultured with naïve CD4^+^ T cells to evaluate their T cell-polarizing capacity. Th2/Th1 polarization was determined based on percentage of T cells staining positive for IL-4/IFN-γ by intracellular staining 10 days later. Data are normalized to DCs pulsed with LPS and represent mean+/−S.E.M. of 4 independent experiments. (**b**) DCs were stimulated with indicated reagents for 6 hours in the presence of neutralizing antibody against DC-SIGN or control antibody after which IL-10 mRNA expression was determined. Expression values are normalized to LPS-stimulated DCs and GAPDH was used as reference gene. Data represent mean+/− S.E.M. of 3 independent experiments. SEA, *S. mansoni* soluble egg antigens; n-ω1, native omega-1 (2 and 0.5 ug/ml); p-ω1, plant-derived omega-1 (8, 2 and 0.5 ug/ml) that carries WT plant N-glycans; p-ω1^H58F^, p-ω1 with H58F mutation in the catalytic site that abrogates RNase activity; p-ω1^LeX^, plant-derived omega-1 that carries the LeX motif.

To evaluate the functionality of plant-produced helminth glycoproteins with different engineered glycans we investigated the immunomodulatory capacity of recombinant omega-1 in a number of functional assays. First we established that omega-1 produced in unmodified *N. benthamiana* (p-ω1) is a functional RNase capable of degrading liver RNA at a protein concentration of 0.5 μg/ml (Fig. S5). This is comparable to native and HEK-cell produced omega-1 under similar assay conditions as described by Everts and co-workers^4^. Next, a strongly increased percentage of IL-4 positive T cells was observed in a co-culture of human T cells with dendritic cells (DCs) that were pre-conditioned with p-ω1 or with LeX-modified recombinant omega-1 from plants (p-ω1^LeX^) in the presence of LPS (Fig. 4a). This Th2 polarizing effect was similar to that of purified native schistosomal omega-1 (n-ω1). In accordance to previous observations using HEK-cell produced omega-1^4^, the Th2 inducing capacity of p-ω1 is dependent on its RNase activity, as p-ω1 with a H58F mutation in the active site of the T2 RNase domain is not capable of Th2 polarisation (Fig. 4a). Previously, the *in vitro* Th2 inducing capacity of omega-1 has also been shown to be dependent on binding to the mannose receptor (MR) on DCs^4^. Binding of omega-1 by MR is facilitated by the plant wild-type paucimannosidic glycans on p-ω1 as well as by the LeX substituted glycans on omega-1 from LeX-engineered plants (p-ω1^LeX^) (Supplemental Fig. 6).

### Functional evaluation of omega-1 from LeX-engineered plants

Natural and synthetic LeX-containing glycoconjugates, including native omega-1 have been shown to induce IL-10 mRNA in DC, in a DC-SIGN-dependent manner (Supplemental Fig. 7)^24^. To evaluate the functional effect of the introduction of LeX on plant-produced omega-1 *in vitro*, we showed that IL-10 mRNA levels were increased significantly in DCs treated with p-ω1^LeX^ compared to p-ω1 (Fig. 4b). Pre-incubation with an anti-DC-SIGN antibody completely inhibited the LeX-mediated elevation of IL-10 mRNA levels. This observation suggests that in addition to the MR-mediated effect observed for p-ω1, the glyco-engineered p-ω1^LeX^ provides an additional DC-SIGN-mediated Th2-related signal to DCs.

**Figure 5:**
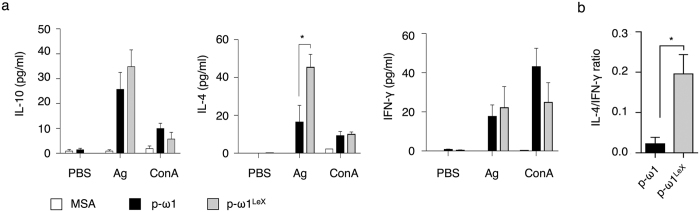
The LeX glycan motif on omega-1 contributes to Th2 polarisation *in vivo.* (**a**) Plant-derived omega-1 glycoforms and mouse serum albumin (MSA) were injected into mouse footpads and 7 days later cytokine responses were determined in draining lymph nodes (LNs) following antigen-specific (Ag) or polyclonal restimulation (ConA) of LN cells *ex vivo*. Cytokine levels were determined in (**a**) supernatants of 3 day restimulated LN cells or (**b**) intracellularly in CD4^+^ T cells following PMA/Ionomycin restimulation. One representative of 2 experiments is shown. Data represent mean+/− S.E.M. of 3 to 4 mice per group (*P < 0.05). p-ω1, plant-derived omega-1 that carries WT plant N-glycans; p-ω1^LeX^, plant-derived omega-1 that carries the LeX motif.

Next, we confirmed that substitution of the LeX motif on the N-glycans of plant-produced omega-1 had functional immunological consequences *in vivo* using a footpad immunization model in mice. Restimulated cells from lymph nodes that drained the site of immunization with p-ω1^LeX^ showed higher expression of the Th2-associated cytokine IL-4, but not IL-10 or IFN-γ, compared to p-ω1 (Fig. 5a). Furthermore, FACS analysis of intracellular cytokine production by CD4^+^ T cells showed a > 5-fold increased ratio of IL-4 over IFN-γ producing cells in response to p-ω1^LeX^ compared to p-ω1 immunization (Fig. 5b, Supplemental Fig. 7). No differences were observed in the total number of lymph node cells and the relative proportion of FoxP3^+^ T cells (Supplemental Fig. 8). Together these data show that engineering of plant-produced omega-1 with LeX-carrying N-glycans similar to the native schistosome glycoprotein strongly increases its Th2 immunomodulatory capacity.

## Conclusion

Our study introduces an alternative for the use of live parasites, crude secretions or recombinant proteins with inappropriate glycans to investigate the immunomodulatory properties of helminths. Inherent to the complex developmental lifecycles of parasitic helminths, purification of substantial amounts of immunomodulatory glycoproteins from helminths is technically not feasible. Furthermore, production in other heterologous expression systems often leads to unintended alterations in the glycan composition. The ability to mimic the immunomodulatory helminth glycome in plants fulfills the increasing demand for helminth glycoproteins by academic and industrial research communities to investigate how helminths are able to dampen host-immune responses. Ultimately, this research could lead to the development of a new class of biopharmaceuticals for the treatment of autoimmune diseases and other chronic inflammatory disorders.

Moreover, helminths infect more than two billion people and are a continuous threat for livestock worldwide, while no vaccines are available for humans, and only scarcely for livestock. The use of *Nicotiana benthamiana* as expression host has already been explored for the production of a hookworm vaccine antigen^25^. Glycoengineering in plants may provide an opening to accelerate the manufacturing of novel vaccines for humans and livestock. This study allows the evaluation of recombinant glycoprotein vaccines with the appropriate glycan structures, which may enhance immunogenicity by promoting correct protein folding, antigen processing and activation of antigen presenting cells.

## Materials and Methods

### Construction of expression vectors

The complete sequences encoding mature *Schistosoma mansoni* omega-1 and kappa-5 proteins were codon optimized in-house. The mature protein sequences were preceded by a signal peptide from the *Arabidopsis thaliana* chitinase gene (cSP). A 6x histidine-FLAG tag (H6F) was included on the C- or N-terminus for omega-1 and kappa-5, respectively. In-house codon optimized genes were synthetically constructed at GeneArt. The H58F mutation in the catalytic site of omega-1 to remove RNase activity was introduced by means of overlap extension PCR. The full sequences were cloned into a pHYG expression vector^26^. Expression vectors (pBIN-PLUS^27^) for hybrid α1,3-fucosyltransferase IXa (Fut9a) from *Tetraodon nigriviridus* (puffer fish) and hybrid β1,4-galactosyltransferase (GalT) from *Danio rerio* (zebrafish) to synthesize LeX structures were used^22^,^23^. The N-terminal CTS domain (C, cytoplasmic tail; T, transmembrane domain; S, stem region) of rat α2,6-sialyltransferase was used to enable *trans*-Golgi targeting (from now on referred to as sialFucT and sialGalT, respectively). For controlled expression of sialGalT we replaced YFP in the pRAP-pGPAII::Gpa2-3′UTR-YFP vector with sialGalT, thereby placing the expression of sialGalT under the control of the promoter region of the *Gpa2* gene^28^. The entire *Gpa2:*sialGalT expression cassette was then transferred to pBIN and pHYG. To synthesize terminal GalNAc residues we cloned the native β1,4-*N*-acetylgalactosaminyltransferase (*bre-4*, referred to as GalNAcT) and the UDP-GalNAc transporters nstp-4, nstp-5 and sqv-7 from *Caenorhabditis elegans* cDNA. A codon optimised C4 epimerase from *Pseudomonas aeruginosa* (WbpP)^17^ was cloned into the pBIN-PLUS expression vector. In all experiments the silencing suppressor p19 from tomato bushy stunt virus in pBIN61 was co-infiltrated to enhance expression^29^.

### Agroinfiltration

*Agrobacterium tumefaciens* (strain MOG101) clones were cultured for 16 hours at 28 °C/250 rpm in LB medium (10 g/L pepton140, 5 g/L yeast extract, 10 g/L NaCl with pH 7.0) containing 50 μg/ml kanamycin and 20 μM acetosyringone. The bacteria were suspended in MMA infiltration medium (20 g/L sucrose, 5 g/L MS-salts, 1.95 g/L MES, pH 5.6) containing 200 μM acetosyringone to a final optical density (OD) of 0.5. For co-infiltration experiments *Agrobacterium* cultures were mixed while maintaining a final OD of 0.5 per culture. The two youngest fully expanded leaves of 5–6 weeks old *Nicotiana benthamiana* plants were infiltrated completely by injecting the *Agrobacterium* suspension into a *N. benthamiana* leaf at the abaxial side using a 1 ml needles syringe. *N. benthamiana* plants were maintained in a controlled greenhouse compartment (UNIFARM, Wageningen) and infiltrated leaves were harvested at 5–6 days post infiltration.

### Protein extraction

For isolation of apoplast proteins, leaves were submerged in ice-cold extraction buffer (50 mM phosphate-buffered saline (pH = 8), 100 mM NaCl and 0.1% v/v Tween-20) after which vacuum was applied for 10 min. Vacuum was released slowly to ensure infiltration of the apoplast. Leaves were placed in 10-ml syringes and centrifuged for 10 min. at 2000 × g. Apoplast fluids were clarified by centrifugation at 16.000 × *g* for 5 min at 4 °C. Remaining intracellular proteins were isolated from the leaves by homogenization in liquid nitrogen. Homogenized plant material was ground in ice-cold extraction buffer (50 mM phosphate-buffered saline (pH = 8), 100 mM NaCl, 0.1% v/v Tween-20 and 2% w/v immobilized polyvinylpolypyrrolidone (PVPP)) using 2 ml/g fresh weight. Crude extracts were clarified by centrifugation at 16.000 × *g* for 5 min at 4 °C. Total protein content was then analyzed by a BCA protein assay (Pierce). Total soluble plant proteins were separated under reducing conditions by SDS-PAGE on a 12% Bis-Tris gel (Invitrogen) and subsequently stained with Coomassie brilliant blue staining.

### Purification from the apoplast fluid

Plant produced omega-1 was purified from the apoplast fluid using HS POROS^®^ 50 strong cation exchange (CEX) resin (Applied Biosystems). In brief, apoplast fluids were transferred over G25 Sephadex columns to exchange for CEX binding buffer (20 mM phosphate buffered saline (pH = 6) containing 100 mM NaCl). Bound omega-1 was eluted with phosphate buffered saline (pH = 6) containing 2 M NaCl. Kappa-5 was purified from apoplast fluids using Ni-NTA Sepharose (IBA Life Sciences) as previously described^14^. Both purifications were performed on an ÄKTA Prime Liquid Chromatography System (GE Healthcare) using a constant flow rate of 2 ml/min. Different samples from the purification of omega-1 or kappa-5 were separated under reducing conditions by SDS-PAGE on a 12% Bis-Tris gel (Invitrogen) and subsequently stained with Coomassie brilliant blue staining.

### Characterization of N*-*glycan composition

The synthesis of LDN structures on plant proteins or kappa-5 was analysed by ELISA with biotinylated agglutinin from soybean (SBA; Vector Labs). For this purpose 10 μg/ml of total soluble apoplast proteins or purified kappa-5 in PBS were coated overnight at 4 °C on ELISA plates. Plates were blocked with PBST containing 1% w/v BSA and all subsequent steps were performed in blocking buffer. Plates were incubated with biotinylated lectin (5 μg/ml) and subsequently avidin-HRP (eBioscience). TMB substrate (eBioscience) was used for detection.

For MALDI-TOF-MS N-glycan analysis, 1–2 μg of purified omega-1 or kappa-5 were reduced and denatured for 10 min at 95 °C in PBS containing 1.3% w/v SDS and 0.1% v/v β-mercaptoethanol. SDS was neutralized by adding 2% v/v NP-40 prior to overnight digestion at 37 °C with trypsin (Sigma-Aldrich) immobilized to NHS-activated Sepharose (GE Healthcare). Trypsin-beads were removed from the digestion mix by centrifugation and the pH of the mix was adjusted to 5 using 1 M sodium acetate. 0.5 mU of PNGase A (Roche) was used to release N*-*glycans from omega-1 while incubating overnight at 37 °C. The incubation mixture was applied to C18 Bakerbond^TM^ SPE cartridges (JT Baker) and the N-glycans were extracted from the flow-through on Extract Clean^TM^ Carbo SPE columns. Eluted N*-*glycans were labeled with anthranilic acid (Sigma-Aldrich) and desalted by hydrophilic interaction chromatography on Biogel P10 (BioRad)^14^. To confirm the presence of LeX in fucosylated N-glycans, the purified N-glycans were treated with α(1–3,4)-fucosidase from *Xanthomonas* sp. (Sigma-Aldrich) and Jack bean β(1–4,6)-galactosidase (Prozyme) according to the suppliers protocols. To confirm the presence of LDN, purified N-glycans were treated with either β-*N*-acetyl-hexosaminidase from *Streptomyces plicatus* (New England Biolabs) or β-*N*-acetyl-glucosaminidase from *Xanthomonas manihotis* (New England Biolabs). Samples in 75% acetonitrile were mixed with 1 μl of matrix solution (20 mg/ml 2,5-dihydroxybenzoic acid in 50% acetonitrile, 0.1% v/v TFA) and were dried under a stream of warm air. Matrix-assisted laser desorption/ionization (MALDI) time-of-flight mass spectra (MS) were obtained using an Ultraflex II mass spectrometer (Bruker Daltonics) as previously described^15^.

### Human dendritic cell-driven T cell polarization assay

Monocytes were isolated from venous blood from healthy volunteers (upon informed consent for participation in this study) according to protocols approved by the Institutional Review Board of Leiden University Medical Center using density centrifugation on Ficoll followed by CD14 + MACS isolation (Miltenyi Biotec) as described previously^4^. Monocytes were differentiated in RPMI medium supplemented with 10% FCS, 20 ng/ml human rGM-CSF (Invitrogen), and 25 U/ml human rIL-4 (R&D Systems). On day 3, culture medium including the supplements was replaced, and on day 6, immature DCs were stimulated with the indicated variants of omega-1 (8, 2, 0.5 μg/ml) in the presence of 100 ng/ml of ultrapure LPS (*Escherichia coli* 0111 B4 strain; InvivoGen). As a Th2 control, DCs were also pulsed with 50 μg/ml of SEA (prepared as described previously^30^,^31^). After 48 hours, DCs were harvested for co-culture with naive T cells. 5 × 10^3^ pulsed DCs were co-cultured with 2 × 10^4^ naive T cells that were purified using a human CD4^+^/CD45RO^−^ column kit (R&D Systems) in the presence of 10 pg/ml staphylococcal enterotoxin B (Sigma-Aldrich) in 96-well flat-bottom plates (NUNC). On day 5, 10 U/ml human rIL-2 (R&D Systems) was added, and the cultures were expanded for another 7 days. For intracellular cytokine production, the primed CD4^+^ T cells were restimulated for 6 hours with 50 ng/ml PMA plus 2 μg/ml ionomycin (both from Sigma-Aldrich). 10 μg/ml brefeldin A was added during the last 2 hours (Sigma-Aldrich). The cells were stained intracellularly for IL-4 (8D4–8) and IFN-γ (25723.11; both from BD Biosciences).

### 3T3-MR binding assay

NIH3T3 cell lines expressing human mannose receptor (3T3.hMR) or the control pFB vector were a kind gift from Joanna Miller and Gordon Brown^32^. The 3T3 cell lines were cultured in DMEM (BioWitthaker) medium + 10% FCS. All media were supplemented with penicillin and streptomycin, and transfected cell lines were continuously kept under selection of 0.5 mg/ml geneticin (Gibco). Cellular adhesion assays were performed as previously reported^33^. Briefly omega-1 glycoforms were fluorescently labeled with PF-647 using the Promofluor labeling kits according to the manufacturer’s recommendations (Promokine). Where indicated cells were pre-incubated with mannan (0.1–1 mg/ml, Sigma-Aldrich) for 45 min at 37 °C prior to addition of fluorescently labeled omega-1 in the stated concentrations at 4 °C. Aliquots of fluorescently labeled p-ω1 and p-ω1^LeX^ glycoforms, treated with α-fucosidase from *X. manihotis* (Sigma-Aldrich) or α-mannosidase from *C. ensiformis* (Sigma-Aldrich) or both, were incubated with 3T3.hMR and control cells to assess the contribution of fucose and mannose residues to MR binding. As control for non-specific binding, cells were incubated with fluorescently labeled BSA at 4 °C. After extensive washes, binding was analysed by flow cytometry (BD FACSAria or FACSCanto, BD Biosciences), using FacsDiva (BD Biosciences) and FlowJo Software (TreeStar).

### *In vivo* experiments

C57BL/6 mice were bred and housed in the animal facility of Leiden University Medical Center and used at 8–12 weeks of age. Mice were bred and/or maintained under specific pathogen–free conditions and all experiments were approved by and conducted in accordance with relevant guidelines and regulations of the institutional animal care body at Leiden University Medical Center. Mice were immunized s.c. into one hind footpad with 10 μg p-ω1, p-ω1^LeX^ or mouse serum albumin (MSA) in 30 μl, and the draining popliteal LNs were analyzed 1 week later. Cells were counted and stained for CD4 (GK1.5), CD25 (PC61.5), CD3 (17A2), FOXP3 (FJK-16s) and CTLA-4 (UC10-4B9) using the FoxP3 staining buffer set (all eBioscience). For antigen specific cytokine responses 1.5 × 10^6^ popliteal LN cells/ml from individual animals were restimulated with medium, 2 μg/ml p-ω1^LeX^ or 10 μg/ml ConA (Sigma-Aldrich) for 4 days in the presence of 5 μg/ml anti-IL-4R (M1; BD Biosciences). Cell culture supernatants were analyzed for IL-10, IL-4 and IFN-γ using the Cytokine Bead Array (BD Biosciences) according to the manufacturer’s recommendation. Assessment of cytokine production by intracellular staining of T cells from these LNs was determined after polyclonal restimulation in 96-well round bottom plates for 6 h with PMA (50 ng/ml) and ionomycin (1 μg/ml) in the presence of Brefeldin A (10 μg/ml) for that last 2 h. The cells were stained with a combination of IL-4 (11B11, eBioscience) and IFN-γ (XMG1.2, BD Biosciences) antibodies and analyzed on a BD Canto II.

### Gene expression analysis

Human dendritic cells were preincubated with 20 μg/ml anti–DC-SIGN (clone AZN-D1; Beckman Coulter) or control antibody for 60 min at 37 °C after which they were stimulated with 100 ng/ml of ultrapure LPS and/or 8 μg/ml of the indicated variants of recombinant omega-1. RNA was extracted from 6 h stimulated DCs using the RNeasy Kit (Qiagen). cDNA synthesis was performed according to standard procedures. Primers were designed using Primer Express (Applied Biosystems) and synthesized by Biolegio. Real-time quantitative PCR was performed using the CFX96 (Biorad). IL-10 mRNA expression levels were normalized by using GAPDH as reference gene.

## Additional Information

**How to cite this article**: Wilbers, R. H. P. *et al*. Production and glyco-engineering of immunomodulatory helminth glycoproteins in plants. *Sci. Rep.* **7**, 45910; doi: 10.1038/srep45910 (2017).

**Publisher's note:** Springer Nature remains neutral with regard to jurisdictional claims in published maps and institutional affiliations.

## Supplementary Material

Supplementary Information
