# Supplementary material for: Production and glyco-engineering of immunomodulatory helminth glycoproteins in plants
Source: Sci Rep. 2017 Apr 10;7:45910. doi: 10.1038/srep45910 (PMC5385521; doi:10.1038/srep45910)
Supplement: Supplementary Information [file srep45910-s1.pdf]

Supplemental Figures belonging to:

Production and glyco-engineering of immunomodulatory helminth glycoproteins in plants

Ruud H. P. Wilbers[a,1,2], Lotte B. Westerhof[a,2], Kim van Noort[a], Katja Obieglo[b], Nicole N. Driessen[b], Bart Everts[b], Sonja I. Gringhuis[c], Gabriele Schramm[d], Aska Goverse[a], Geert Smant[a], Jaap Bakker[a], Hermelijn H. Smits[b], Maria Yazdanbakhsh[b], Arjen Schots[a,3] & Cornelis H. Hokke[b,1,3]

[a] Laboratory of Nematology, Plant Sciences Group, Wageningen University and Research, Droevendaalsesteeg 1, 6708 PB Wageningen, The Netherlands

[b] Department of Parasitology, Leiden University Medical Center, Albinusdreef 2, 2333 ZA Leiden, The Netherlands

[c] Department of Experimental Immunology, Academic Medical Center, University of Amsterdam, Meibergdreef 9, 1105 AZ Amsterdam, the Netherlands

[d] Research Center Borstel, Priority Area Asthma and Allergy, Experimental Pneumology, Parkallee 22, D-23845, Borstel, Germany.

[1] Corresponding authors

[2] Equal contribution

[3] Joint senior authors

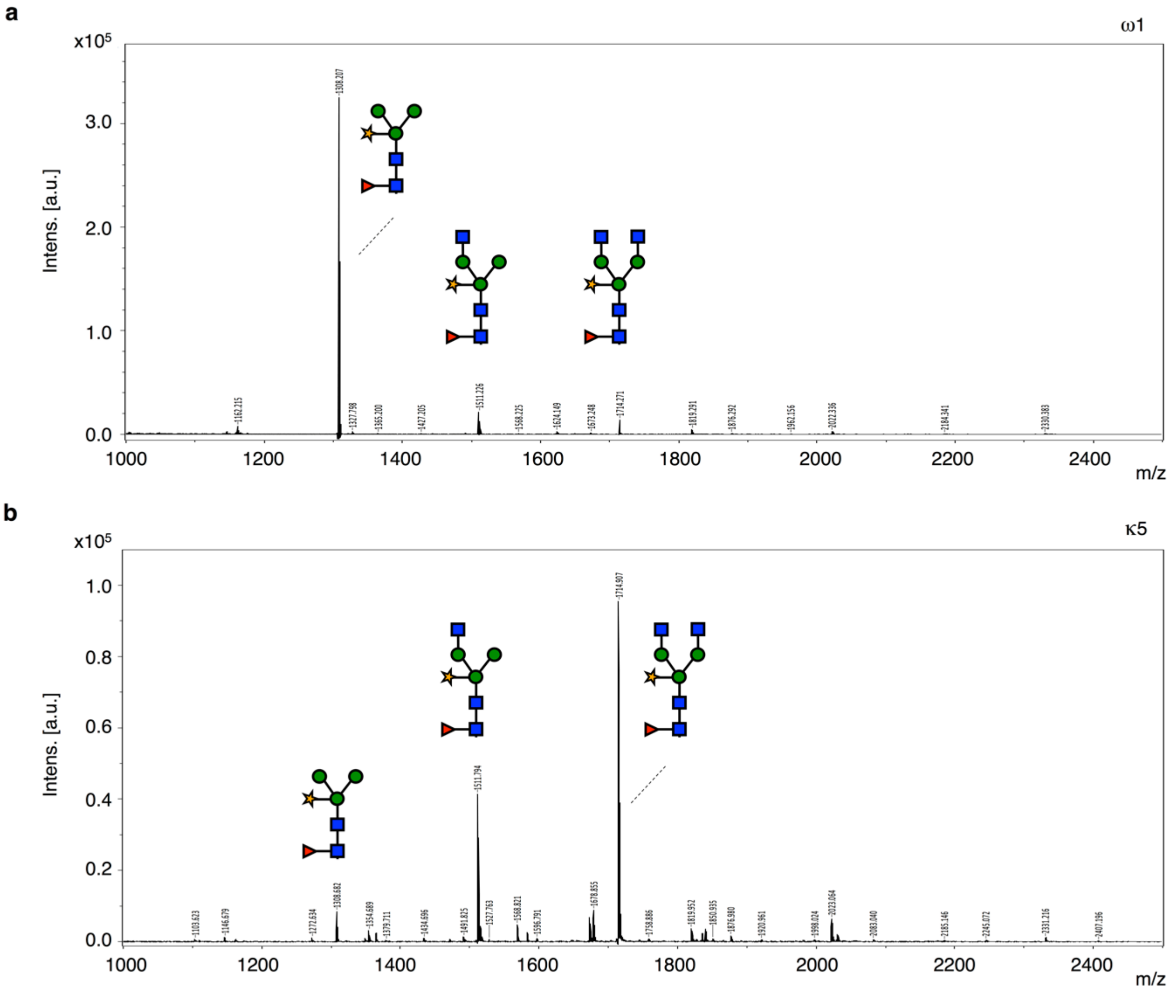

Supplemental figure 1. N-glycan profiles for omega-1 and kappa-5 upon expression in *N. benthamiana* plants. N-glycan composition of omega-1 (**a**) and kappa-5 (**b**) purified from apoplast was analyzed using MALDI-TOF-MS. The predominant N-glycans on both proteins carry β1,2-xylose and core α1,3-fucose. However, omega-1 terminal residues are mannose (paucimannosidic), whereas kappa-5 N-glycans terminate in GlcNAc residues. The lack of terminal GlcNAc residues on omega-1 likely arises from a protein specific sensitivity towards β-N-acetylhexosaminidase activity in the apoplast.

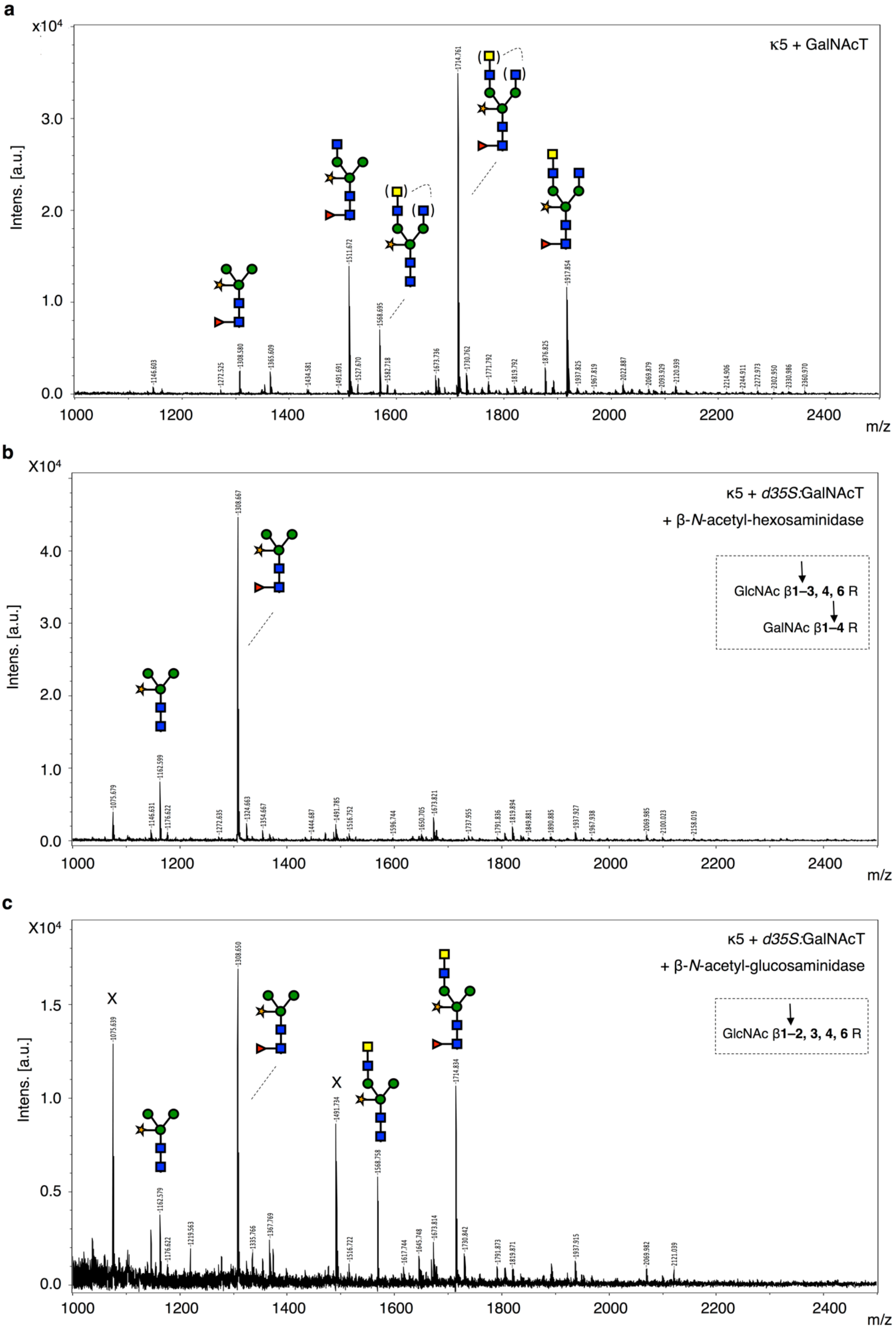
a
x10⁴
Intens. [a.u.]
κ5 + GalNAcT
m/z
b
X10⁴
Intens. [a.u.]
κ5 + d35S:GalNAcT
+ β-N-acetyl-hexosaminidase
GlcNAc β1–3, 4, 6 R
GalNAc β1–4 R
m/z
c
X10⁴
Intens. [a.u.]
κ5 + d35S:GalNAcT
+ β-N-acetyl-glucosaminidase
GlcNAc β1–2, 3, 4, 6 R
X
X
m/z

Supplemental figure 2. Confirmation of the presence of LDN structures. N-glycans from purified kappa-5 were analysed by MALDI-TOF-MS. (**a**) N-glycan profile for kappa-5 from wild-type *N. benthamiana* plants upon co-expression of GalNAcT. (**b**) Profile of the same N-glycans upon treatment with β-*N*-acetyl-hexosaminidase from *Streptomyces plicatus*. (**c**) Profile of the same N-glycans upon treatment with β-*N*-acetyl-glucosaminidase from *Xanthomonas manihotis.* For both enzymatic digestions the substrate specificity is indicated in the dashed box. β-*N*-acetyl-glucosaminidase digestion reveals the successful synthesis of LDN. Sugar residues for which the position is not clear (prior to enzymatic digestion) are placed between brackets.

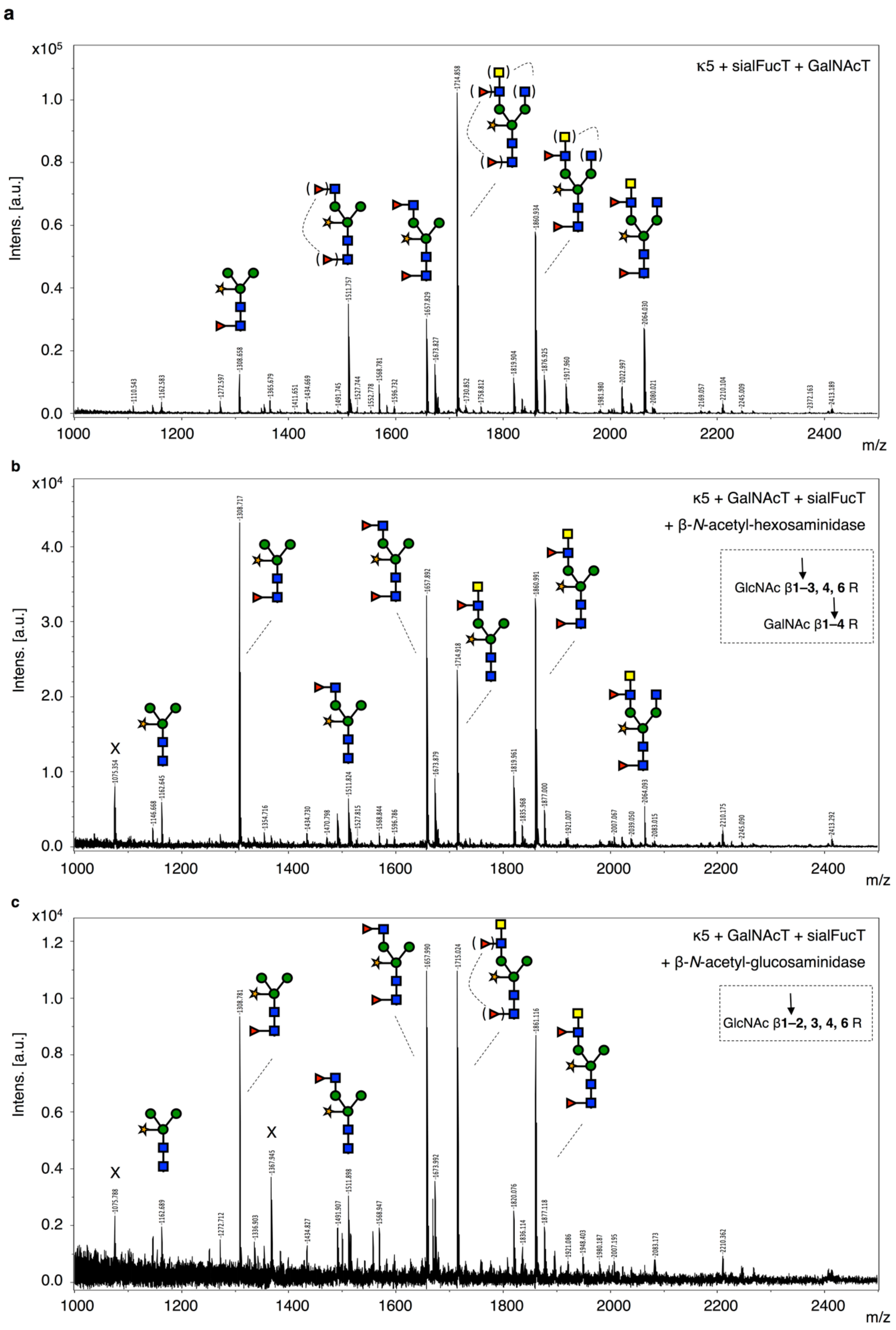
a
x10^5
Intens. [a.u.]
m/z
κ5 + sialFucT + GalNAcT
b
x10^4
κ5 + GalNAcT + sialFucT
+ β-N-acetyl-hexosaminidase
GlcNAc β1–3, 4, 6 R
GalNAc β1–4 R
c
κ5 + GalNAcT + sialFucT
+ β-N-acetyl-glucosaminidase
GlcNAc β1–2, 3, 4, 6 R

Supplemental figure 3. Confirmation of the presence of LDN-F structures. N-glycans from purified kappa-5 were analysed by MALDI-TOF-MS. (**a**) N-glycan profile for kappa-5 from wild-type *N. benthamiana* plants upon co-expression of GalNAcT and sialFucT. (**b**) Profile of the same N-glycans upon treatment with β-*N*-acetyl-hexosaminidase from *Streptomyces plicatus*. (**c**) Profile of the same N-glycans upon treatment with β-*N*-acetyl-glucosaminidase from *Xanthomonas manihotis.* For both enzymatic digestions the substrate specificity is indicated in the dashed box. β-*N*-acetyl-glucosaminidase digestion and partial β-*N*-acetyl-hexosaminidase digestion reveals the successful synthesis of LDN-F. Sugar residues for which the position is not clear (prior to enzymatic digestion) are placed between brackets.

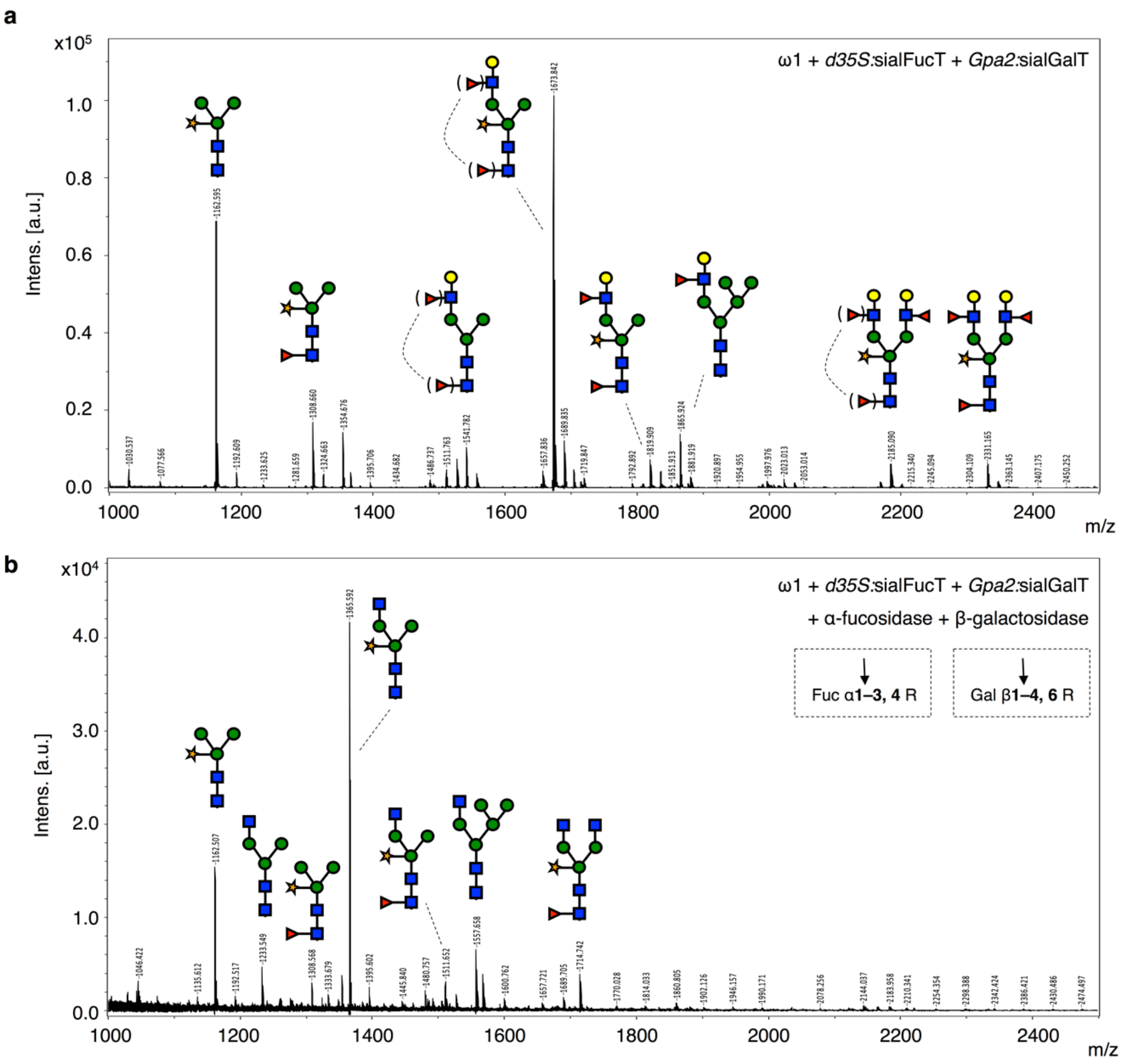

Supplemental figure 4. Confirmation of the presence of LeX glycan motifs. N-glycans carrying LeX motifs on purified omega-1 were analysed by MALDI-TOF-MS. (**a**) N-glycan profile for omega-1 from wild-type *N. benthamiana* plants upon co-expression of *d35S*:sialFucT and *Gpa2*:sialGalT, but the latter being expressed with the pHYG vector. (**b**) Profile of the same N-glycans upon treatment with α(1-3,4)-fucosidase from *Xanthomonas* sp. (cleaves LeX associated fucose) and β(1-4,6)-galactosidase from Jack bean. For enzymatic digestion the substrate specificity is indicated in the dashed box. Sugar residues for which the position is not clear (prior to enzymatic digestion) are placed between brackets.

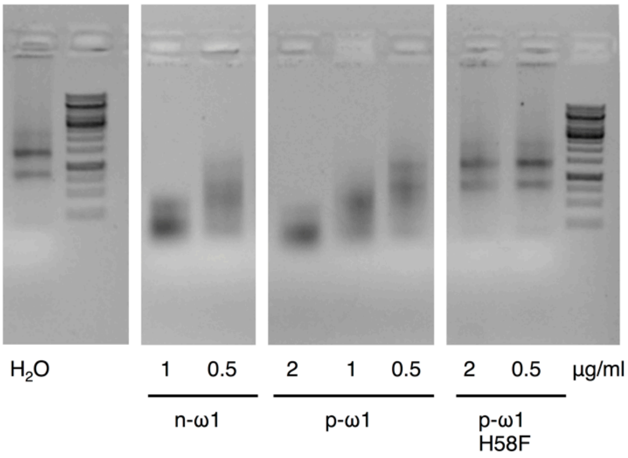

Supplemental figure 5. RNase activity of plant-produced omega-1. Purified omega-1 from plant apoplast fluids (p-ω1 and p-ω1^{H58F}) were compared to natural omega-1 (n-ω1) for their ability to degrade total mouse liver RNA. RNA degradation was assessed by agarose gel electrophoresis. Both n-ω1 and p-ω1 have similar RNase activity, whereas p-ω1^{H58F} is not active due to the mutation in the active site of the T2 RNase domain.

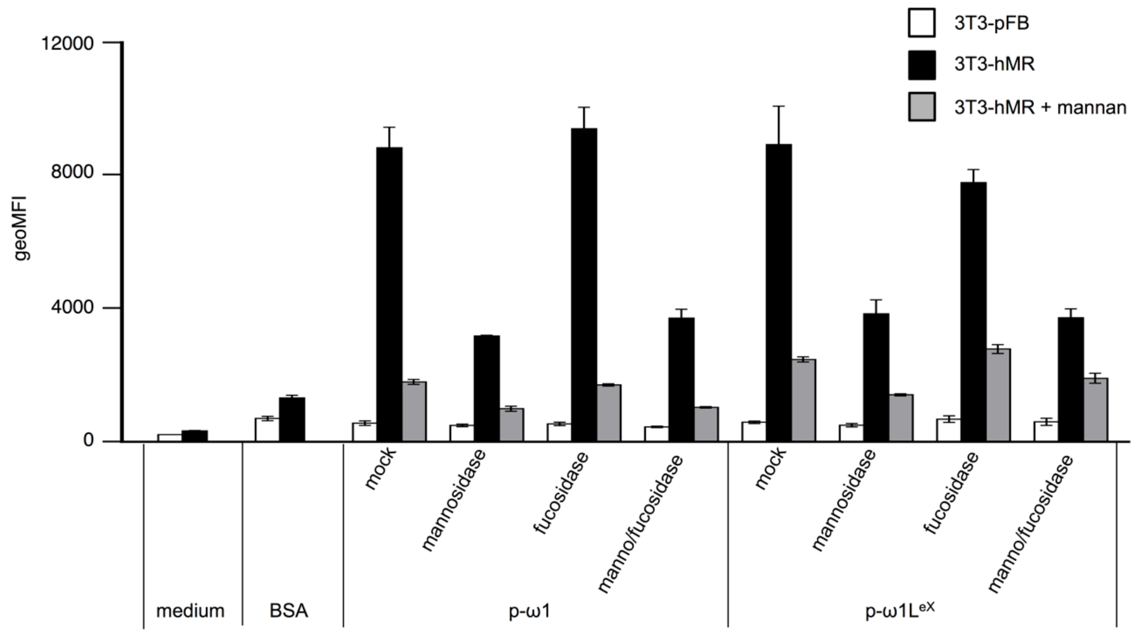

Supplemental figure 6. Plant-produced omega-1 binds to MR independent of the presence of LeX glycan motifs. MR-transfected 3T3 cells were incubated with fluorescently-labelled p-ω1 and p-ω1^{LeX}, either mock treated, α-mannosidase treated to remove terminal mannose residues, α-fucosidase treated to remove LeX-associated fucose, or treated with both enzymes. MR-specificity was checked in all cases by pre-treatment with mannan. p-ω1 binding decreases after mannosidase treatment, but not after fucosidase treatment, whereas p-ω1^{LeX} binding to MR decreased after both treatments indicating the both terminal mannose and LeX-associated fucose facilitate MR binding of omega-1, in line with the N-glycan structures indicated in **Supplemental Fig 1a, 4a.** The experiments were performed in duplicate and one representative experiment out of two is shown. Error bars represent S.D.

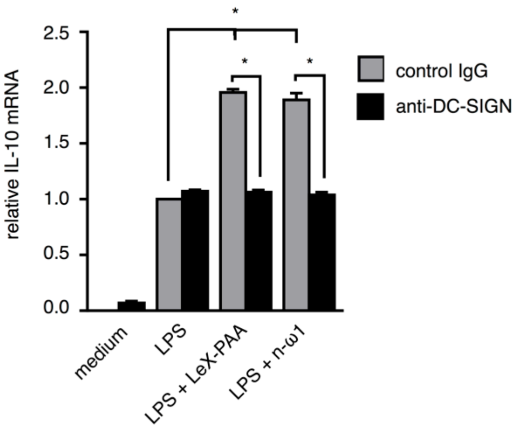

Supplemental figure 7. Induction of IL-10 expression by native omega-1 and Lewis X. Human monocyte-derived dendritic cells (DCs) were stimulated with LPS and a synthetic Lewis X conjugate (LeX-PAA) or native omega-1 (n-ω1) for 6 hours in the presence of neutralizing antibody against DC-SIGN or control antibody after which IL-10 mRNA expression was determined. Expression values are normalized to LPS-stimulated DCs and GAPDH was used as reference gene. Data represent mean +/- S.E.M. of 3 independent experiments.

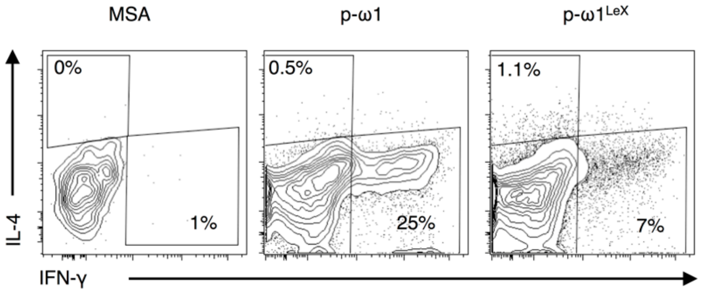

Supplemental figure 8. Th2 priming capacity of plant-produced omega-1. Plant-derived omega-1 glycoforms and mouse serum albumin (MSA) were injected into mouse footpads and 7 days later cytokine responses were determined in draining lymph nodes (LNs) following antigen specific or polyclonal restimulation of LN cells *ex vivo*. IL-4 and IFN-γ expression was determined in $CD4^+$ T cells by intracellular staining following PMA/Ionomycin restimulation. Representative FACS plots are given corresponding to the ratio IL-4-IFN-γ positive T cells given in **Figure 5d**.

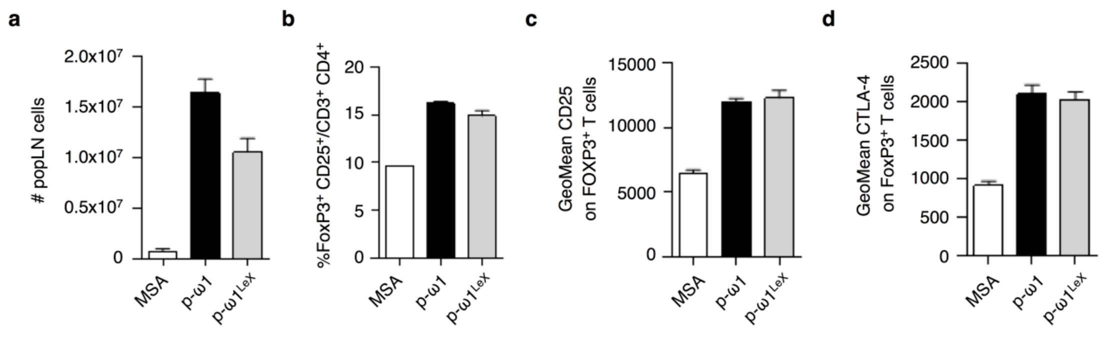

Supplemental figure 9. Presence of LeX glycan motifs on omega-1 does not change its Treg inducing potential. Plant-derived omega-1 glycoforms were injected into mouse footpads and 7 days later draining LNs were analyzed for (**a**) cell number, (**b**) frequency of $FoxP3^+$ Tregs within CD4 T cell compartment, (**c**) CD25 and (**d**) CTLA4 expression on $FoxP3^+$ Tregs. One representative of 2 experiments is shown. Data represent mean +/- S.E.M. of 3 to 4 mice per group (*$P < 0.05$).
